# Hippocampal CA1 Pyramidal Neurons of *Mecp2* Mutant Mice Show a Dendritic Spine Phenotype Only in the Presymptomatic Stage

**DOI:** 10.1155/2012/976164

**Published:** 2012-07-30

**Authors:** Christopher A. Chapleau, Elena Maria Boggio, Gaston Calfa, Alan K. Percy, Maurizio Giustetto, Lucas Pozzo-Miller

**Affiliations:** ^1^Department of Neurobiology, Civitan International Research Center, The University of Alabama at Birmingham, Birmingham, AL 35294, USA; ^2^Department of Neuroscience, University of Torino and National Institute of Neuroscience, Turin 10126, Italy; ^3^Neuroscience Institute, CNR, Pisa 56125, Italy; ^4^IFEC-CONICET and Department of Pharmacology, School of Chemical Sciences, Cordoba National University, Cordoba 5000, Argentina; ^5^Department of Pediatrics, The University of Alabama at Birmingham, Birmingham, AL 35294, USA

## Abstract

Alterations in dendritic spines have been documented in numerous neurodevelopmental disorders, including Rett Syndrome (RTT). RTT, an X chromosome-linked disorder associated with mutations in *MECP2*, is the leading cause of intellectual disabilities in women. Neurons in *Mecp2*-deficient mice show lower dendritic spine density in several brain regions. To better understand the role of MeCP2 on excitatory spine synapses, we analyzed dendritic spines of CA1 pyramidal neurons in the hippocampus of *Mecp*2^*tm*1.1Jae^ male mutant mice by either confocal microscopy or electron microscopy (EM). At postnatal-day 7 (P7), well before the onset of RTT-like symptoms, CA1 pyramidal neurons from mutant mice showed lower dendritic spine density than those from wildtype littermates. On the other hand, at P15 or later showing characteristic RTT-like symptoms, dendritic spine density did not differ between mutant and wildtype neurons. Consistently, stereological analyses at the EM level revealed similar densities of asymmetric spine synapses in CA1 *stratum radiatum* of symptomatic mutant and wildtype littermates. These results raise caution regarding the use of dendritic spine density in hippocampal neurons as a phenotypic endpoint for the evaluation of therapeutic interventions in symptomatic *Mecp2*-deficient mice. However, they underscore the potential role of MeCP2 in the maintenance of excitatory spine synapses.

## 1. Introduction

The postsynaptic sites of excitatory glutamatergic synapses in the brain, dendritic spines, are small protrusions that extend from dendrites and are associated with structural plasticity [1]. While the role of dendritic spines in models of learning and memory and synaptic plasticity continues to gain support, alterations in dendritic spine density and morphology have been consistently documented in numerous disorders associated with intellectual disabilities [2, 3]. One such disease associated with both intellectual disability and dendritic spine pathology is Rett syndrome (RTT; MIM 312750). RTT is an X chromosome-linked disorder that affects approximately 1 : 10,000–15,000 females worldwide and is the leading cause of severe intellectual disabilities in females [4]. In addition to a reduction in the size of neuronal cell bodies, a decrease in dendritic complexity of pyramidal cells was described in several brain regions [5–7]. Previous work in our laboratory using postmortem brain tissue from female RTT individuals demonstrated that hippocampal CA1 pyramidal neurons have lower spine density than age-matched female unaffected individuals [8]. Similar qualitative observations had previously been reported in pyramidal neurons of the motor cortex [9].

Mutations in *MECP2*, the gene encoding methyl-CpG-binding protein-2, have been identified in ~90% of RTT individuals [10–12]. Using an *in vitro* organotypic slice culture system, we demonstrated that expression of *MECP2* missense mutations commonly found in RTT individuals caused a significant reduction of dendritic spine density in hippocampal pyramidal neurons, especially of the more mature mushroom type spines [8]. Consistent with these findings, neurons generated *in vitro* from induced pluripotent stem cells (iPSCs) derived from skin fibroblasts of RTT patients showed lower dendritic spine density than control neurons [13]. While these observations shed light on the role of MeCP2 in the formation and/or maintenance of excitatory synapses on dendritic spines, further characterizing the available mouse models of RTT will allow defining phenotypic endpoints to evaluate novel pharmacological interventions.

The most studied mouse models of RTT either lack the MeCP2 protein by deletion of *Mecp2* exons 3 and 4 (Bird line) [14] or express a nonfunctional mutant protein due to deletion of *Mecp2* exon 3 (Jaenisch line) ([15]; reviewed in [16, 17]). Quantitative confocal microscopy of Lucifer yellow-labeled neurons from both of these *Mecp2*-deficient mouse strains revealed lower dendritic spine density in several brain regions, including pyramidal neurons of the CA1 region of the hippocampus [18]; it should be noted that these observations were made in postnatal-day 21 mice (before overt RTT-like symptoms), and that they are consistent with the lower density of excitatory synapses estimated from VGLUT1-PSD95 puncta observed in the hippocampal CA1 region of 2-week-old *Mecp*2^*tm*1.1Bird^ null mice [19]. Symptomatic *Mecp*2^*tm*1.1Bird^ null mice at 5 weeks, however, showed comparable density of VGLUT1-PSD95 puncta to that in wildtype littermates [19].

To better understand the role of *Mecp2* on dendritic spine maintenance, we analyzed dendritic spine density and morphology by confocal microscopy and excitatory synapses by electron microscopy in hippocampal CA1 pyramidal neurons of presymptomatic (P7 and P15) and symptomatic (P40–60) male *Mecp*2^*tm*1.1Jae^ mutant mice and their age-matched wildtype littermates [15]. Our observations indicate that dendritic spine density is lower only at postnatal-day 7 (P7), while it does not differ at P15 or later when symptoms are well established. Consistently, stereological analyses at the EM level revealed comparable density of asymmetric spine synapses between symptomatic *Mecp2 *mutant and wildtype littermates. These results raise caution regarding the use of dendritic spine density in hippocampal neurons as a phenotypic endpoint for the evaluation of therapeutic interventions in symptomatic *Mecp2*-deficient mice. In addition, these observations demonstrate that proper *Mecp2* function is required for the early development of dendritic spines in CA1 pyramidal neurons and that a secondary compensatory mechanism seems to take place in symptomatic *Mecp2 *mutant mice.

## 2. Materials and Methods

### 2.1. Animals

Breeding pairs of mice lacking exon 3 of the X chromosome-linked *Mecp2* gene (B6.Cg-*Mecp*2^tm1.1Jae^, Jaenisch strain; C57BL/6 background) [15] were purchased from the Mutant Mouse Regional Resource Center at UCDavis. A colony was established at UAB by breeding wildtype males with heterozygous *Mecp*2^*tm*1.1Jae^ mutant females, following the original breeding scheme [15], which is recommended by the supplier. Genotyping was performed by PCR of sample DNA from tail clips. Hemizygous male mice of the *Mecp*2^*tm*1.1Jae^ mutant strain are healthy until 5-6 weeks of age, when they begin acquiring RTT-like motor symptoms, such as hypoactivity, hind limb elevation, and reflex impairments [15]. For the present studies, the experimental subjects were homozygous *Mecp*2^*tm*1.1Jae^ mutant males (called *Mecp2* mutants) and wildtype male mice that were littermates of postnatal-day 7 (P7), P15, and between P40 and P60. Animals were handled and housed according to the Committee on Laboratory Animal Resources of the National Institutes of Health. All experimental protocols were annually reviewed at The University of Alabama at Birmingham and at The Università di Torino and approved by each institution's respective Institutional Animal Care and Use Committee.

### 2.2. DiOlistic Labeling

Mice that were postnatal-day (P7) were euthanized by cervical dislocation, and the brain was immersed in 4% paraformaldehyde (in 0.1 M phosphate buffer); hippocampi were later dissected and kept in fixative for an additional 2 hours. P15 or P40–60 mice were deeply anesthetized with ketamine (100 mg/kg) and euthanized by transcardiac perfusion with ~200 mL of 4% paraformaldehyde (in 0.1 M phosphate buffer).

**Figure 1:**
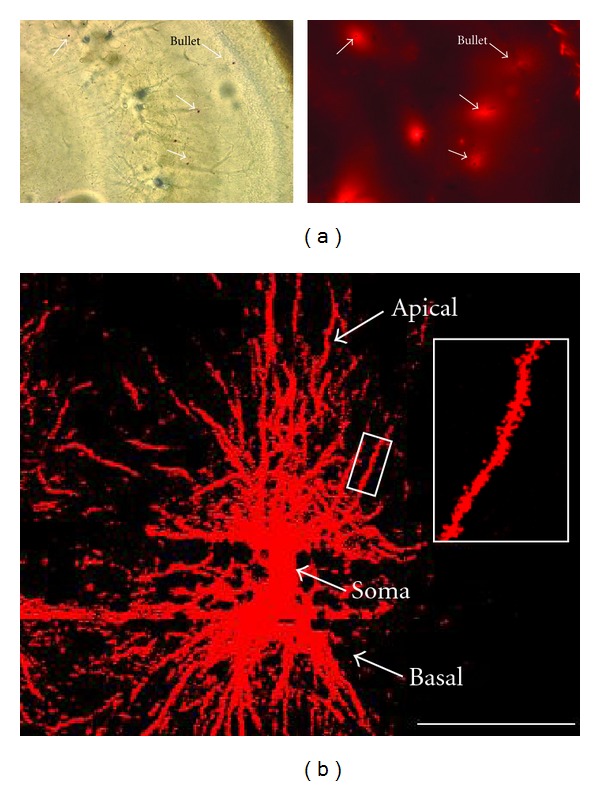
Dendritic spines in the CA1 region of the hippocampus visualized by DiI “DiOlistics” and confocal microscopy. (a) (left), Brightfield image of the CA1 region of a representative formalin-fixed hippocampal section (100 *μ*m thickness) stained with DiI by particle-mediated labeling (DiOlistics) showing tungsten bullets used to deliver DiI (arrows). (right), DiI fluorescence from the same field of view. (b) Representative CA1 pyramidal neuron stained with DiI and imaged by confocal microscopy. Inset: apical dendritic segment representative of those selected for quantitative analyses of dendritic spines.

After dissection, brains and hippocampi were rinsed several times in 0.1 M phosphate buffered saline (PBS) and cut into 100 *μ*m thick coronal sections with a McIlwain tissue chopper, which were further rinsed in 0.1 M PBS and stored at 4°C until DiI labeling. To visualize dendritic spines by laser-scanning confocal microscopy, coronal sections containing the hippocampus were stained with the lipophilic fluorescent dye 1,1′-dioctadecyl-3,3,3′,3′-tetramethylindocarbocyanine perchlorate (DiI; InVitrogen, Carlsbad, CA, USA) by particle-mediated labeling (DiOlistics) [20, 21]. First, DiI was diluted in dimethyl chloride (methylene chloride; Sigma, St. Louis, MO, USA). Then, 20 mg of 1.1 *μ*m tungsten particles (Bio-Rad; Hercules, CA, USA) were placed on top of a pre-cleaned glass slide and spread out evenly with two pre-cleaned razor blades. The DiI solution was added onto the tungsten particles and allowed to completely evaporate. To prevent clumping of the DiI/tungsten mixture, razor blades were used to break apart the mixture. Additionally, a small amount of polyvinylpyrrollidone (10–20 *μ*L PVP made in fresh in 100% ethanol; Bio-Rad) was added to the DiI/tungsten mixture to further prevent particle clumping and improve their coating to the Tefzel tubing. The DiI-coated tungsten particles were then added to a glass tube with 3 mL of water and sonicated for 1 hr. After sonication, the solution was vortexed and then aspirated and coated onto Tefzel tubing for 15 mins. After 15 mins, the solution was removed and the tubing was allowed to dry for 15 mins. DiI-coated tungsten bullets were shot only once onto individual hippocampal slices with a custom-modified Helios hand-held gene gun (Bio-Rad) using 75 psi He pressure through a 40 *μ*m pore size filter [8]. After labeling with DiI bullets, slices were rinsed and stored in PBS for 15–30 min at room temperature in the dark to allow diffusion of DiI. Then, slices were postfixed with 4% paraformaldehyde and stored at 4°C overnight. Slices were finally washed with PBS and mounted on glass slides with Vectashield (Vector Laboratories, Burlingame, CA, USA).  Figure 1(a) shows tungsten bullets and the resulting DiI fluorescence in a representative section of perfusion-fixed hippocampus stained by DiOlistics. It should be noted, from examining Table 1, for mice that were younger in age, the total dendritic length examined is smaller than that obtained for the older mice. This discrepancy is presumably a result of older mice having an abundant number of cells and with longer dendrites.

### 2.3. Laser-Scanning Confocal Microscopy

High-resolution images of spiny apical secondary or tertiary dendrites showing adequate DiI labeling were acquired from CA1 *stratum radiatum* in a Fluoview FV-300 laser-scanning confocal microscope (Olympus, Center Valley, PA, USA) using an oil immersion 60 X 1.45 NA objective lens (PlanApo, Olympus), with additional  3x digital zoom. DiI was excited with an HeNe Green laser (543 nm), and its fluorescence detected with a cube containing a 555 nm dichroic mirror and a 585 ± 40 nm emission filter (Semrock, Lake Forest, IL, USA). Series of optical sections in the *z*-axis were acquired with 0.1 *μ*m intervals through individual apical dendritic branches.  Figure 1(b) shows representative examples of a CA1 pyramidal neuron and a segment of apical dendrite stained with DiI.

### 2.4. Analysis of Dendritic Spine Density

Dendritic spines were identified as small protrusions that extended less than 3 *μ*m from the parent dendrite and counted manually in maximum-intensity projections of confocal *z*-stacks using ImageJ software (W. S. Rasband, ImageJ, US National Institutes of Health, Bethesda, Maryland, USA; http://rsb.info.nih.gov/ij/, 1997–2009); protrusions that were more than 3 *μ*m were classified as filopodia and rarely seen except in the P7 mice. Care was taken to ensure that each spine was counted only once by following its projection course through the stack of z-sections. Spines were counted only if they appeared continuous with the parent dendrite. Spine density was calculated by quantifying the number of spines per dendritic segment and normalized to 10 *μ*m of dendrite length. Microscope calibrations were performed using 1.07 *μ*m fluorescent latex microspheres (Molecular Probes, Eugene, OR, USA), which yielded a lateral resolution of 0.09 *μ*m per pixel. Counting and measuring of individual spines was conducted in a blind fashion, as the genotype was unknown to the person performing the image analysis.

### 2.5. Dendritic Spine Classification

The categorization of different morphological spine types was performed as described [22, 23]. Briefly, geometrical dimensions of individual spines were measured in maximum-intensity projections of the *z*-stacks using ImageJ and used to calculate the L/N and H/N ratios, where L is spine length, H is the maximum head width, and N is the maximum neck width. Then, spine types were grouped as mature-shaped spines, which included type-I (stubby) and type-II (mushroom) shaped spines, or immature-shaped thin (type-III) spines, following published criteria [24]. Table 1 shows the spine density and proportions of spine types in each genotype, as well as the total number of dendritic spines counted and measured and the total dendritic length analyzed.

### 2.6. Electron Microscopy and Stereological Synapse Count

Brain fixation and preparation for electron-microscopy analyses were performed as described [25]. Male *Mecp*2^*tm*1.1Jae^ mutant mice and wildtype littermates (3 per group) were anaesthetized with an intraperitoneal injection of chloral hydrate and transcardially perfused with ice cold 2% paraformaldehyde and 2.5% glutaraldehyde in 0.1 M phosphate buffer (PB; pH 7.4). After perfusion, brains were left in the same fixative overnight at 4°C, washed several times in 0.1 M PB, and then postfixed with 1% osmium tetroxide (in 0.1 M cacodylate buffer) for 1 hour. For resin embedding, tissues were dehydrated in a graded series of ethanol (30–100%) and infiltrated with an Epon-Araldite mixture. Ultrathin serial sections (70 nm) were cut with an ultramicrotome (Leica Ultracut, Wetzlar, Germany) and collected on single slot copper grids coated with a pioloform solution. The grids were counterstained with uranyl acetate and lead citrate and imaged in a JEM-1010 electron microscope (Jeol, Japan) equipped with a side-mounted CCD camera with 1376 × 1032 pixels (Mega View III; Soft Imaging System GmbH, Muenster, Germany). Asymmetric synapses were identified by the presence of at least three vesicles within the profile adjacent to the presynaptic membrane and the presence of a clear PSD. Unbiased stereological estimation of asymmetric spine synapses (presumptive excitatory synapses) was performed as previously described [26] by using the double dissector method [27]. In this method, images of two serial sections are acquired, the first of which is designated as the “reference section” and the second as the “look-up section.” Twenty-five pairs of nonoverlapping electron micrographs were acquired for each animal in CA1 *stratum radiatum* at ×20,000 magnification. Within an unbiased counting frame that represented 28 *μ*m^2^, the number of synapses that were present in the reference section but absent in the look-up section were counted. The cross-section area of dendritic spines and presynaptic terminals was analyzed on the same digital images using ImageJ as previously described [26]. At least 180 synapses were analyzed per genotype.

### 2.7. Statistical Analyses

Averages of multiple measurements are presented as mean ± standard error of the mean (SEM). Data were statistically analyzed using unpaired Student's *t*-test using Prism software (GraphPad Software, Inc., San Diego, CA, USA). Probability values lower than 0.05 were considered statistically significant (i.e., *P* < 0.05, less than 5% probability that the observations are due to chance). When lower than this cut-off value, the actual *P* values are given in Section 3 (rather than just the statement “greater than” or “less than”) to provide readers with more detailed information regarding the outcome of the statistical analyses. Cumulative frequencies plots were first analyzed using the normal distribution Kolmogorov-Smirnov (K-S) fitting test, and then K-S two-sample tests for subsequent paired comparisons.

## 3. Results

### 3.1. Presymptomatic *Mecp2* Mutant Mice: CA1 Pyramidal Neurons Have Lower Spine Density Only in P7, While It Does Not Differ at P15

We previously showed that pyramidal neurons from rat hippocampus overexpressing human *MECP2* carrying single point missense mutations commonly found in RTT individuals have lower dendritic spine density than control neurons [8]. Consistent with lower dendritic spine density in female RTT individuals, such spine loss in mutant *MECP2*-expressing neurons was observed after 4 days of over expression *in vitro* in organotypic slice cultures. In addition, 3-week-old *Mecp2*-deficient mice have lower spine density than age-matched wildtypes [18], but older symptomatic *Mecp2* mutant mice show comparable density of VGLUT1-PSD95 puncta to that in wildtype littermates [19]. To address a potential developmental progression of this dendritic spine phenotype in* Mecp2* mutant mice (*Mecp*2^*tm*1.1Jae^), we performed quantitative analyses of dendritic spines in DiI-labeled hippocampal sections from mutant and age-matched wildtype littermates by laser-scanning confocal microscopy.

**Figure 2:**
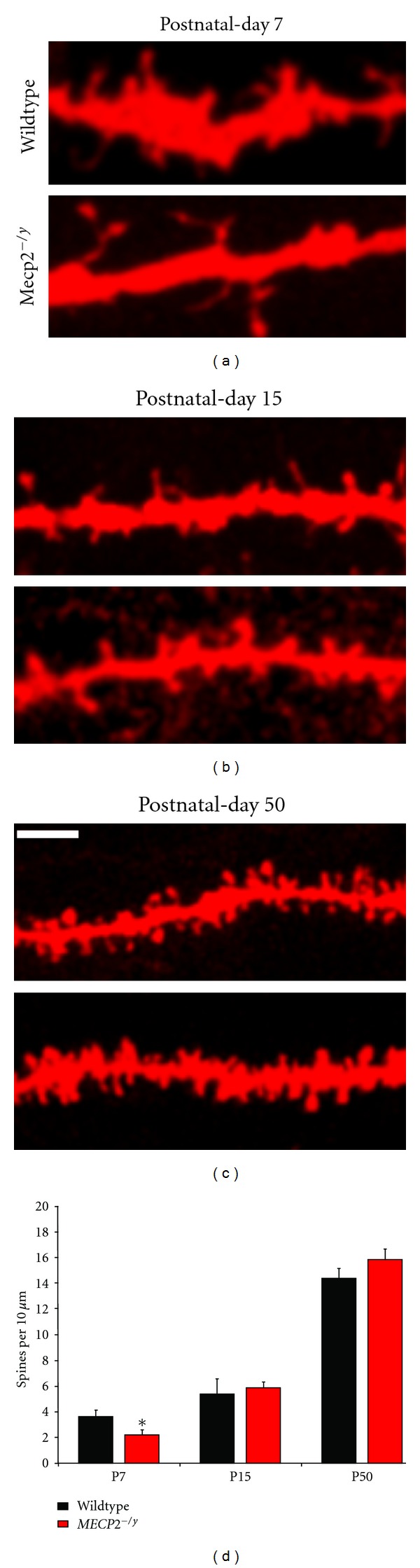
Quantitative analyses of dendritic spines in CA1 pyramidal neurons from *Mecp2* mutant mice and age-matched wildtype littermates. (a) Representative examples of apical dendritic segments of CA1 pyramidal neurons from P7 wildtype and *Mecp*2^*tm*1.1Jae^ mice (top). Scale bar represents 2 *μ*m.(b) Examples of apical dendritic segments of CA1 pyramidal neurons from P15 wildtype and *Mecp*2^*tm*1.1Jae^ mice (top). (c) Examples of apical dendritic segments of CA1 pyramidal neurons from P40 wildtype and *Mecp*2^*tm*1.1Jae^ mice (top). (d) Dendritic spine density (spines per 10 *μ*m of dendrite). All data are expressed as mean ± SEM. *indicates *P *< 0.05.

Figure 2(a) shows representative examples of maximum-intensity projections of confocal stacks from segments of secondary apical dendrites of CA1 pyramidal neurons used for quantitative analyses. Dendritic spine density in CA1 pyramidal neurons of P7 *Mecp*2^*tm*1.1Jae^ mutants was lower compared to age-matched wildtype littermates (wildtype 3.65 ± 0.54 spines per 10 *μ*m of dendritic length, *n* = 5 dendrite segments from 3 mice, versus *Mecp*2^*tm*1.1Jae^ 2.23 ± 0.40 spines/10 *μ*m, *n* = 10 segments/3 mice; *P* = 0.030; Figures 2(a) and 2(d)). On the other hand, dendritic spine density in P15 *Mecp*2^*tm*1.1Jae^ mutant mice was not statistically different compared to age-matched wildtype littermates (wildtype 5.39 ± 1.20 spines/10 *μ*m, *n* = 7 segments/3 mice versus *Mecp*2^*tm*1.1Jae^ 5.87 ± 0.43 spines/10 *μ*m, *n* = 9 segments/3 mice; *P* = 0.359; Figures 2(b) and 2(d)).

Despite differences in spine density in P7 mice, the proportions of the three main morphological types of spines did not differ between *Mecp*2^*tm*1.1Jae^ and age-matched wildtype littermates (stubby: wildtype 0.49 ± 0.07 versus *Mecp*2^*tm*1.1Jae^ 0.46 ± 0.08; *P* = 0.396, mushroom: wildtype 0.24 ± 0.02 versus *Mecp*2^*tm*1.1Jae^ 0.16 ± 0.04; *P* = 0.110, thin: wildtype 0.27 ± 0.05 versus *Mecp*2^*tm*1.1Jae^ 0.25 ± 0.06; *P* = 0.401). Similarly, the proportions of morphological spine types were comparable in P15 mice of both genotypes (stubby: wildtype 0.39 ± 0.01 versus *Mecp*2^*tm*1.1Jae^ 0.33 ± 0.03; *P* = 0.087, mushroom: wildtype 0.47 ± 0.03 versus *Mecp*2^*tm*1.1Jae^ 0.50 ± 0.03; *P* = 0.244, thin: wildtype 0.14 ± 0.03 versus *Mecp*2^*tm*1.1Jae^ 0.16 ± 0.02; *P* = 0.269). 

### 3.2. CA1 Pyramidal Neurons of Symptomatic *Mecp2* Mutant Mice Have Comparable Dendritic Spine Density and Morphology Than Their Age-Matched Wildtype Littermates

Dendritic spine density in CA1 pyramidal neurons of P40–60 *Mecp*2^*tm*1.1Jae^ mutant mice exhibiting characteristic RTT-like symptoms were not statistically different than in age-matched wildtype littermates (wildtype 14.39 ± 0.77 spines/10 *μ*m of dendritic length, *n* = 35 segments/5 mice versus *Mecp*2^*tm*1.1Jae^ 15.82 ± 0.86 spines/10 *μ*m, *n* = 29 segments/5 mice; *P* = 0.110; Figures 2(c) and 2(d)). We next determined if the proportion of the three major morphological types of dendritic spines were affected in symptomatic *Mecp*2^*tm*1.1Jae^ mutant mice. The proportion of stubby spines and mushroom spines, larger spines considered to be more stable, was comparable between the genotypes (stubby: wildtype 0.51 ± 0.02 versus *Mecp*2^*tm*1.1Jae^ 0.53 ± 0.02; *P* = 0.211; mushroom: wildtype 0.33 ± 0.01 versus *Mecp*2^*tm*1.1Jae^ 0.35 ± 0.01; *P* = 0.06). On the other hand, the proportion of the more motile and immature thin spines was lower in symptomatic *Mecp*2^*tm*1.1Jae^ mutant mice (wildtype 0.16 ± 0.01 versus *Mecp*2^*tm*1.1Jae^ 0.11 ± 0.01; *P* = 0.005).

### 3.3. The Density and Morphology of Asymmetric Spine Synapses in CA1 *Stratum radiatum* of Symptomatic *Mecp2* Mutant Mice Is Comparable to That of Their Age-Matched Wildtype Littermates

**Figure 3:**
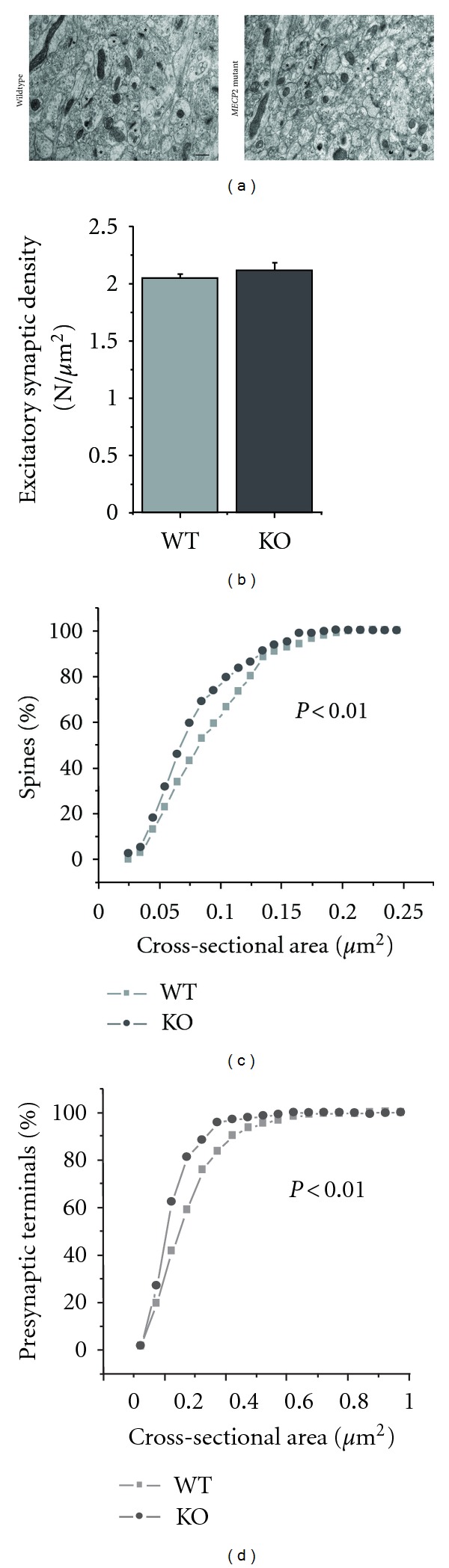
Quantitative electron microscopic analyses of CA3-CA1 excitatory synapses in the hippocampus of symptomatic *Mecp*2^*tm*1.1Jae^ mutant mice and age-matched wildtype littermates. (a) Electron micrographs of *stratum radiatum* in the CA1 area of the hippocampus from wildtype and symptomatic male *Mecp*2^*tm*1.1Jae^ mutant mice (P56). Representative excitatory synapses are indicated (asterisks). Scale bar: 400 nm. (b) Histograms show the number per unit of volume of asymmetric/excitatory CA3-CA1 synapses in CA1 *stratum radiatum*. The analysis using stereology (dissector method) revealed that *MECP2* mutation does not affect excitatory synapse number. (c) Cumulative percentage of the cross-sectional area of dendritic spines associated with asymmetric CA3-CA1 synapses. Area of the dendritic spine was determined when associated with a presynaptic terminal. (d) Cumulative percentage of the cross-sectional area of presynaptic terminals associated with asymmetric CA3-CA1 synapses. Area of the presynaptic terminals was determined when associated with a dendritic spine.

Electron microscopy of asymmetric synapses on dendritic spines (presumptive excitatory) within CA1 *stratum radiatum* of symptomatic *Mecp*2^*tm*1.1Jae^ mutant mice was conducted to determine if *Mecp2* alters density of excitatory synapses. Ultrastructural observations revealed a phenotype consistent with the above confocal microscopy results (Figure 3(a)). Unbiased stereological analyses revealed that the density of asymmetric spine synapses in symptomatic *Mecp*2^*tm*1.1Jae^ mutant mice (2.12 ± 0.11 synapses per *μ*m^2^; *n* = 3 mice) was not significantly different than in wildtype littermates (2.06 ± 0.05 synapses/*μ*m^2^, *n* = 3 mice; *P* = 0.4; Figure 3(b)). Intriguingly, the cross-sectional area of individual spines (Figure 3(c)) and presynaptic terminals (Figure 3(d)) were smaller in *Mecp*2^*tm*1.1Jae^ mutant mice (Kolmogorov-Smirnov test; *P* < 0.01), but only for the smallest asymmetric spine synapses. It should be noted that these spines have dimensions below the resolution of diffraction-limited light microscopy and thus are not detectable in our dendritic spine density measurements relying on confocal microscopy (Figure 2(b)).

## 4. Discussion

Quantitative confocal microscopy of DiI-labeled dendrites revealed that CA1 pyramidal neurons from P7 *Mecp2* mutant mice had lower spine density compared to age-matched wildtype littermates. These differences in spine density were not observed neither in slightly older but still presymptomatic *Mecp2* mutant mice at P15 nor in P40–60 mice that express the full spectrum of RTT-like symptoms, consistent with an analysis of the density of VGLUT1-PSD95 puncta in area CA1 [19]. Stereological analyses at the electron microscopy level revealed that the density of asymmetric spine synapses is comparable in P40–60 fully symptomatic *Mecp*2^*tm*1.1Jae^ mutant mice compared to age-matched wildtype littermates. The only significant difference between genotypes during the symptomatic stage was a lower proportion of immature thin spines and a smaller cross-sectional area of individual spines and presynaptic terminals—but only for the smallest asymmetric spine synapses—in *Mecp*2^*tm*1.1Jae^ mutant mice. It should be noted that the smallest asymmetric spine synapses analyzed at the EM level have dimensions below the resolution of diffraction-limited light microscopy and thus are not detectable in our measurements using confocal microscopy. Our observations during the symptomatic stage demonstrate that *Mecp2* loss-of-function causes subtle structural modifications of excitatory CA3-CA1 synapses without major changes in excitatory synapse density, as we showed in an independent EM study using random single ultrathin sections [28]. These data demonstrate that proper *Mecp2* function is required for early development of dendritic spines in CA1 pyramidal neurons and that a compensatory mechanism that normalizes spine density seems to occur later in development.

A recurrent theme in cytological studies of postmortem RTT brains is the observation of significant differences in the fine structure of dendrites. Studies of neurons from cortical regions of the brain have shown impaired dendritic branching in individuals with RTT [5]. In addition, qualitative observations of pyramidal neurons of the motor cortex of RTT individuals described segments of dendrites that were bare of spines [9]. Using quantitative confocal microscopy of DiI-labeled hippocampal sections, we recently showed that CA1 pyramidal neurons have lower dendritic spine density in female RTT individuals than in age-matched unaffected females [8]. Our results on hippocampal dendritic spine density show that male *Mecp*2^*tm*1.1Jae^ mutant mice at the symptomatic stage do not recapitulate the human phenotype observed in autopsy material from female RTT individuals. While many factors could contribute to these findings (including gender differences, disease severity, seizure disorder, and X-chromosomal inactivation ratio), these results suggest that hippocampal neuron spine density in this particular mouse model is not a phenotype with sufficient face validity for RTT. However, these mice still yield relevant information on the role of *Mecp2* in the CNS. Further postmortem studies in more individuals with RTT as well as other *MECP2*-associated conditions are needed to better understand the consequences on dendritic spine density and morphology. If such studies are correlated with a detailed clinical history, they will further yield a better understanding of the contribution of other factors to the spine phenotype, such as specific *MECP2* mutations, disease severity and progression, and life-long medications.

Our previous *in vitro* studies in rat hippocampal neurons in primary culture demonstrated that either shRNA-mediated knockdown of endogenous MeCP2 protein levels or overexpression of human *MECP2* missense mutations common in RTT patients (R106W or T158M) reduced dendritic length and branching during early neuronal development [29]. Furthermore, using a postnatal rat hippocampal slice culture preparation, we observed that the knockdown of endogenous MeCP2 protein levels resulted in reduced dendritic spine density, especially of mature spines; however, overexpression of RTT-associated human *MECP2 *missense mutations led to a dramatic reduction in dendritic spine density [8]. Consistently, a recent report described that neurons derived from iPSCs obtained from reprogramming of skin fibroblasts of RTT patients have reduced dendritic spine density [13]. While these exciting observations on patient-derived neurons provide additional evidence of the importance of dendritic spines in the neuropathology of RTT, it should be made clear that no causative biochemical underpinning has yet been established.

Transgenic mice that either lack *Mecp2* or express a mutant nonfunctional *Mecp2* peptide fragment are excellent experimental models to help determine the synaptic defects that contribute to RTT. These mice recapitulate several behavioral features of RTT and display many defects in dendritic structure and synaptic transmission and plasticity (reviewed in [16, 17]). However, they have also yielded varying results in terms of dendritic spine alterations. In contrast with the present observations, a quantitative study of two different *Mecp2* mutant lines (*Mecp*2^*tm*1.1Bird^ and *Mecp*2^*tm*1.1Jae^) described that pyramidal neurons from hippocampal area CA1 and layers II-III of the motor cortex have lower dendritic spine densities than their control wildtype littermates at approximately 3 weeks of age [18]. The parsimonious explanation for this apparent discrepancy is that these studies used mice of different strains and the genetic background of the different mouse lines contributed to the divergent observations on dendritic spine density. While both studies employed commercially available *Mecp*2^*tm*1.1Jae^ mice, the genetic background is different: we used *Mecp*2^*tm*1.1Jae^ mice on a pure C57BL/6 background inbred for more than 10 generations, while Belichenko et al. [18] used two different lines (*Mecp*2^*tm*1.1Jae^ and *Mecp*2^*tm*1.1Jae^) on a mixed genetic background [18]. Genetic background has been known to contribute significantly to several biological parameters. For example, neurite outgrowth is significantly different in two mouse strains where *Nogo-A* was knocked out [30]. When comparing two different *Mecp2* transgenic mouse lines maintained on different genetic backgrounds, Belichenko et al. [18] described significant differences in spine density between age-matched wildtype mice of the two different strains, strongly suggesting that genetic background contributes to the phenotype under study, for example, dendritic spine density and morphology [18].

We show here that hippocampal pyramidal neurons exhibit a dendritic spine phenotype only in neonatal (P7) mutant mice, well before excitatory synapse expansion, while spine density in mutants recovers to wildtype levels a week later (P15) and is maintained at wildtype levels throughout the symptomatic stage (P40–60). This developmental progression of the dendritic spine phenotype is also reflected in the density of VGLUT1-PSD95 puncta, which is lower in area CA1 of 2-week-old *Mecp2* null mice, but comparable to wildtype levels at 5 weeks of age [19]. Together with our dendritic spine observations, those VGLUT1-PSD95 puncta results are consistent with the present EM analyses in symptomatic *Mecp*2^*tm*1.1Jae^ mutant mice, which revealed comparable densities of asymmetric spine synapses in *stratum radiatum* of area CA1 of both genotypes [28]. Altogether, these data demonstrate that proper *Mecp2* functioning is required for dendritic spine formation during early postnatal development, and that a secondary compensatory mechanism seems to take place in symptomatic *Mecp2 *mutant mice. A couple of possibilities exist as to the extent of the compensatory mechanisms necessary to bring spine density to wildtype levels. One possibility is that enhanced hippocampal network activity in *Mecp2* mutants promotes dendritic spine formation [28]. A second possibility is that deranged homeostatic plasticity promotes spinogenesis, while still affecting pyramidal neuron function [31].

Despite the lack of differences in dendritic spine density in fully symptomatic *Mecp2* mutant mice, we observed a significant, yet small, reduction in the proportion of immature thin spines in *Mecp2* mutants compared to age-matched wildtype animals. What consequence this might have on hippocampal synaptic function remains debatable; however, it is hypothesized that thin spines represent “learning spines” because of their constant changing in response to neuronal activity (e.g., LTP and LTD), while mushroom spines are considered more mature and stable “memory spines” [32]. Considering that dendritic spines are highly sensitive to the levels of neuronal activity [33], the shift in the proportion of morphological spine types could also reflect a response to the heightened neuronal activity observed in the hippocampal network of symptomatic *Mecp*2^*tm*1.1Jae^ mutant mice [28]. Thus, it would be interesting to determine what role *Mecp2* has in the maintenance of thin spines and what consequences does this have on hippocampal function. One report has already demonstrated that spine motility is slowed in *Mecp2* mutant mice [34], possibly reflecting the decrease in the proportion of thin spines that we observed.

Confocal microscopy of dendritic spines in organotypic hippocampal cultures has revealed that approximately 65–70% of dendritic spines are juxtaposed to presynaptic terminals [35]. For this reason, we decided to conduct unbiased stereological analyses at the electron microscopy level to determine the density and morphology of dendritic spines that were actually connected to a presynaptic terminal, for example, asymmetric spine synapses. This approach demonstrated that the density of asymmetric spine synapses in *Mecp*2^*tm*1.1Jae^ mutant mice is comparable to that of wildtype littermates, consistent with confocal microscopy of dendritic spines. Intriguingly, the areas of dendritic spines and presynaptic terminals are smaller in *Mecp*2^*tm*1.1Jae^ mutant mice; however, this difference was only observed for the smallest asymmetric spine synapses. Considering that these spines have dimensions below the resolution of diffraction-limited light microscopy, they could not have been included in measurements of spine head width in confocal microscopy images (Figure 3(b)). Taken together, our observations demonstrate that while the proportion of thin spines is lower in *Mecp*2^*tm*1.1Jae^ mutant mice, individual dendritic spines, and thus excitatory synapses, are smaller in volume.

In summary, our results raise caution regarding the use of dendritic spine density in hippocampal neurons as a phenotypic endpoint for the evaluation of therapeutic interventions in symptomatic *Mecp2* deficient mice. However, we present data describing the importance of *Mecp2* on spine development in neonatal mice. Future research will hopefully explain the precise molecular role of MeCP2 in the establishment of the hippocampal excitatory network and how this manifest into clinical issues.

## Figures and Tables

**Table 1:** Total number of mice, length of dendrites, and individual dendritic spines counted and measured in the quantitative analyses.

Genotype	Mice	Total length of dendrites (*μ*m)	Total number of spines
P7 wildtype	3	463.96	168
P7 *Mecp2 * ^−/*y*^	3	994.81	235
P15 wildtype	3	647.92	303
P15 *Mecp2 * ^−/*y*^	3	676.06	385
P40–60 wildtype	5	2,107.02	2,931
P40–60 *Mecp2 * ^−/*y*^	5	2,131.24	3,478
